# Snake venom toxins as potential therapeutic agents in the treatment of prostate cancer

**DOI:** 10.1007/s11033-024-09970-z

**Published:** 2024-11-14

**Authors:** Jesika Ochoa-Mosquera, Alejandro Montoya-Gómez, Eliécer Jiménez‑Charris

**Affiliations:** Grupo de Nutrición, Facultad de Salud, Universidad del Valle, Calle 4B # 36–00, Edificio 116, Oficina, Cali 5002 Colombia

**Keywords:** Venom molecules, Bioprospecting, Drug discovery, Anticancer agent, Prostatic carcinoma

## Abstract

Prostate cancer is a significant global health concern and one of the leading causes of death from diseases in men. There is a growing interest in exploring new therapeutic approaches to enhance patient treatment outcomes and quality of life. Snake venom-derived compounds have emerged as promising candidates for anticancer treatment due to their potential to be selective and reduce adverse effects. In this article, we conduct a literature review on prostate cancer and discuss the investigation of snake venoms as potential alternatives in treatments to minimize toxicity and maximize efficacy. The potential of snake venom toxins in modulating key processes such as cell apoptosis, inhibition of cell migration, and angiogenesis is highlighted. This comprehensive exploration reaffirms the importance of advancing research into snake venom-based therapies to combat prostate cancer, transform treatment paradigms, and improve the well-being of affected individuals.

## Introduction

Cancer constitutes one of the leading causes of mortality in the world and represents a significant challenge in increasing life expectancy worldwide. The Global Cancer Observatory (GLOBOCAN) registered approximately 19.9 million new cases of cancer and approximately 9.7 million deaths from cancers globally. Among the most prevalent types of cancer among the male population, PCa is one of the most commonly diagnosed, together with lung cancer, colorectal cancer, and stomach cancer [1]. Worldwide, during the year 2022, PCa was the second most common cancer (1,467,854 new cases), accounting for 14.2% of the reported cancer diagnoses, and ranked as the fifth highest cause of mortality in men (397,430 deaths), accounting for 7.3% [2]. These numbers are expected to increase over time. According to GLOBOCAN, an increase in diagnoses close to 80% is estimated for the year 2050, and deaths will increase by 30%, reaching approximately 516,314 deaths [3]. In Colombia, according to statistics for the incidence of cancer in both sexes for the year 2022, PCa is the second most diagnosed cancer after breast cancer and is the leading cancer detected in men, with a total of 16,479 identified cases and 4,304 deaths from this neoplasia, ranking as the fifth highest cause of cancer mortality in Colombia [4].

Current treatments for PCa include surgery, radiation therapy, chemotherapy, and hormonal therapies. These treatments have been designed to address the different stages and characteristics of PCa, and the approach used depends on the cancer stage, the person’s age, and general health [5]. However, these therapies can generate significant side effects in patients, such as erectile dysfunction and urinary incontinence, caused by a lack of tumor environment selectivity [6].

In this context, scientific research plays a crucial role in the search for new treatments that are more selective. In particular, efforts have been made to explore natural molecules with therapeutic potential, such as those derived from snake venoms. Various investigations have explored the anticancer potential of snake venoms and the proteins that compose them, and studies have indicated that these molecules interfere with critical processes in tumor progression, such as migration, cell invasion, and angiogenesis [7–13]. Although the influence of snake venoms and their constituent molecules on various types of neoplasms has been documented, to date, no review has focused on the effects of snake venoms and their constituent molecules on prostate cancer models. For this reason, through a review of scientific works, in this article, we compile existing evidence to propose molecules from snake venoms as possible pharmacological candidates for treating PCa.

## Natural history of prostate cancer

**Fig. 1:**
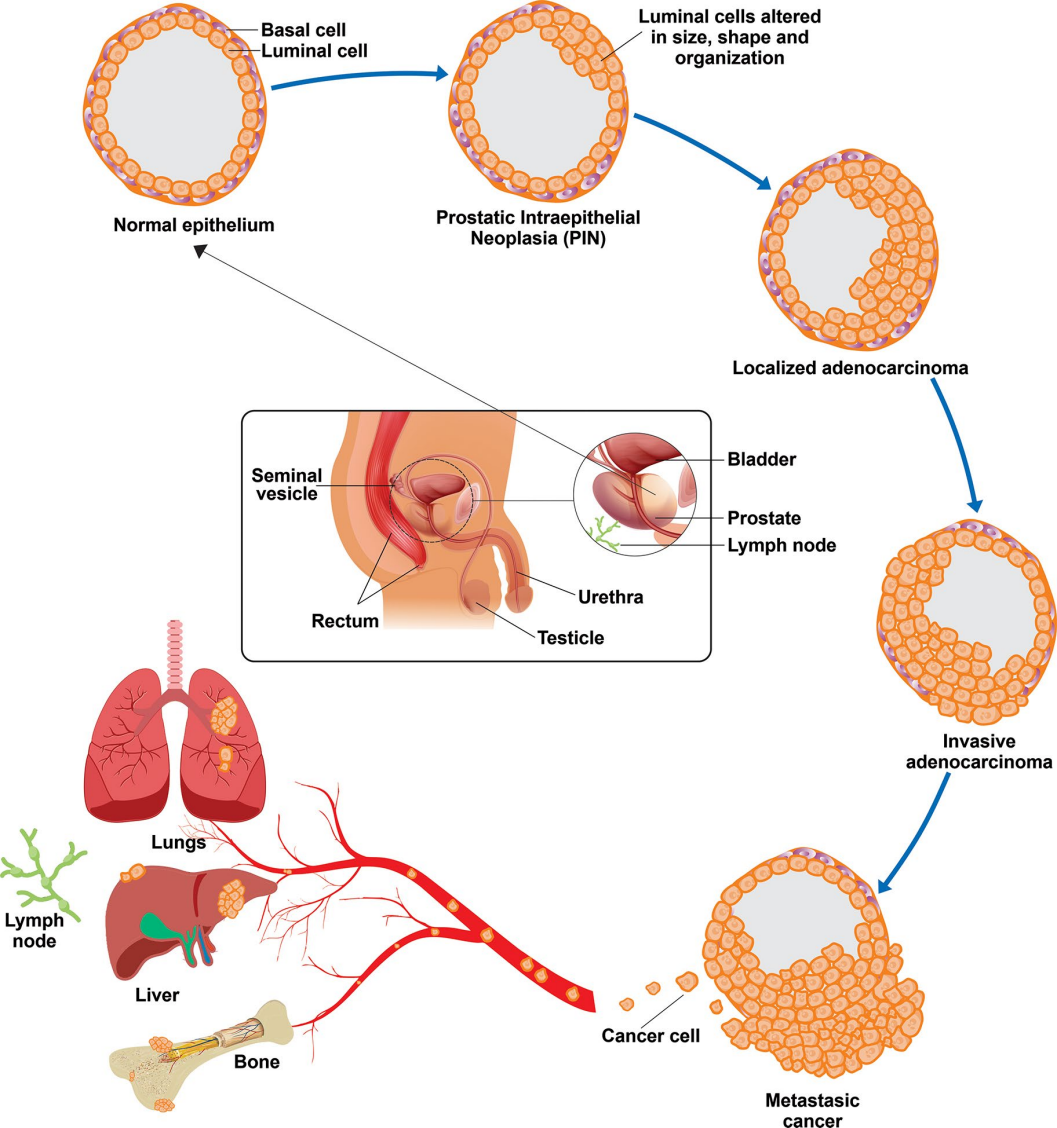
Illustrative diagram of the prostate anatomy and formation of malignant tumors. The initiation, progression, and advancement of prostate cancer are linked to the activation of proto-oncogenes and the inhibition of tumor suppressor genes. Abnormalities in the prostate epithelium lead to pre-neoplastic lesions known as prostatic intraepithelial neoplasia (PIN), characterized by luminal epithelial hyperplasia and decreased basal cells. PIN lesions can evolve into invasive adenocarcinoma (luminal phenotype) with the loss of the basal cell layer and basement membrane, resulting in various tumor grades. These grades range from indolent to more aggressive forms of PCa, eventually leading to metastasis to primary target organs such as the lungs, liver, bone, and lymph nodes

The prostate gland is part of the male reproductive system, just below the bladder and in front of the rectum. It is similar in shape to a walnut and surrounds the urethra (Fig. 1). The prostate’s primary function is to produce prostatic fluid, which is a crucial part of semen and essential for sperm viability and motility [14].

Prostate cancer (PCa) occurs when prostate gland cells start to grow uncontrollably, leading to the formation of malignant tumors (Fig. 1). In over 70% of PCa cases, the tumors develop in the peripheral part of the prostate (the outer part of the gland). They are predominantly of adenocarcinoma histological type, with less frequent occurrences of squamous cell carcinomas. Between 50% and 90% of cases are characterized as multifocal with local progression, spreading to the iliac, pelvic obturator, and sacral nodes through the lymphatic system, eventually involving bone tissue in metastasis [14, 15]. PCa is a heterogeneous disease with different histomorphological and molecular characteristics, presenting itself in various ways due to different causal factors in each affected man. As a result, at the clinical level, it can range from an asymptomatic localized tumor to rapidly progressing metastatic PCa [16].

The progression of PCa from the initial stage as a primary cancer to developing into metastatic disease involves several phases. Initially, inflammatory, overgrowing, and highly proliferative lesions may be generated, considered precursors of PCa. These lesions, known as prostatic intraepithelial neoplasms (PIN), show cellular stratification, crowding, and irregularities in the basal cell layer. As it progresses, the tumor cells acquire more genetic mutations, allowing them to grow and spread more aggressively. PIN can evolve into noninvasive carcinoma in situ, the precursor to locally invasive prostatic adenocarcinoma [17].

The majority of primary prostate cancers are multifocal and exhibit significant genomic diversity, leading to variation among affected individuals. Even within the same patient, there can be variability within tumors and between tumors [16]. These characteristics complicate the clinical management and staging of the disease. The progression of prostate cancer involves its spread to nearby organs, such as the bladder or rectum, and may cause symptoms such as difficult or painful urination and blood in the urine. At this stage, prostate cancer can also become resistant to treatments such as hormone therapy, resulting in metastatic prostate cancer. Cancer cells can then spread from the primary tumor to adjacent lymph nodes through the bloodstream or lymphatic system, reaching distant organs such as the liver, lungs, and, most commonly, the bones, leading to severe pain, hypercalcemia, and frequent fractures [18]. Several factors are associated with the development and progression of prostate cancer, as described below.

### Risk factors

#### Hormonal factors

Several factors, including hormonal factors, influence the development of PCa. An essential component in the prostate is the prostatic acinus, which has glandular elements that allow the production and secretion of Prostate Specific Antigen (PSA) and acid phosphatase, a protein involved in the dissolution of the seminal clot and in sperm motility [19]. The activity of these epithelial cells is influenced by androgenic action [20, 21], with testosterone being the hormone with the most significant influence on the growth and maturation of the prostatic epithelium [22]. The development of the prostatic epithelium is dependent on a reduction process that occurs in the glandular cells, where testosterone is reduced at the 4,5 double bond, giving the formation of dihydrotestosterone, by the action of the enzymes 5-α-reductase type 1 and 2 (5αR1 and 5αR2). This new component forms a complex with the androgen receptors (AR) that can interact with specific sequences of the DNA chain, promoting the growth, survival, and production of PSA [21]. Dysregulated upregulation of this complex causes alterations that can lead to sustained survival and growth signals, eventually leading to the development of PCa. Increased expression of 5αR1 and 5αR2 hormones has been demonstrated in advanced PCa, and increased 5αR1 in PIN and PCa, compared to benign prostatic hyperplasia (BPH), which favors the hypothesis of dependence on dysregulation of these enzyme isoforms for tumor progression [20].

#### Genetic factors

PCa is conditioned to several genetic alterations that can lead to the formation of abnormal prostate cells, activating oncogenes and deactivating tumor suppressor genes, leading to uncontrolled proliferation of malignant cells. The main alterations are related to changes in the AR, PTEN/PIK3CA, BRCA1 and 2, and HOXB13 genes, among many others. Individuals can acquire these genetic mutations throughout life or by family predisposition [23–25]. A family history of PCa and other types of neoplasms increases the probability of mutations in genes such as HOXB13, BRCA1 and 2, MMR, which are associated with PCa at an early age [25].

AR contributes to controlling the expression of specific genes in response to androgen binding, such as testosterone [25]. Most mutations in AR result in one amino acid substitution located in the androgen binding site; this point mutation is involved in resistance to hormone therapy [24]. For example, the T878A mutation has been identified in the PCa cell line LNCaP; this alteration allows the RA to be activated not only by androgens such as testosterone but also by other steroids present in the body such as progesterone, estrogen, and anti-androgens such as cyproterone, which would not usually activate the receptor, allowing the proliferation of abnormal cells to continue despite hormonal treatment [26, 27]. Another common mutation is H875Y, which correlates with a lack of response to enzalutamide therapy in patients previously treated with abiraterone and docetaxel [26]. In addition, this substitution has been found in CWR22 prostatic carcinoma cells and is associated with resistance to hormonal treatments [27]. Finally, the F876L mutation transforms antagonists such as enzalutamide into agonists, facilitating the progression of castration-resistant cancer. The cancer cell line LNCaP has been identified as the T878A and F876L substitutions that co-occur [27]. There has also been evidence of structural rearrangements in the AR gene in metastatic PCa tumors, resulting in AR variants devoid of the ligand-binding domain so that their activation is androgen-independent. An example is the AR-V7 variant, associated with PCa progression and its resistance to castration [28, 29].

The PTEN tumor suppressor gene exerts its action by inhibiting the PI3K/AKT/mTOR signaling pathway, which regulates biological functions such as cell growth and survival [25]. In PCa, mutations or deletions of the PTEN gene have been demonstrated to generate a loss of its function, promoting over-activation of the PI3K/AKT/mTOR pathway, generating uncontrolled cell proliferation, resistance to programmed cell death, and stimulation of angiogenesis [24, 25, 30, 31]. The PTEN gene mutation could be used as a diagnostic genomic marker to differentiate an indolent tumor from an aggressive one [31].

The BRCA1 and BRCA2 genes are tumor suppressor genes involved in DNA repair and have been associated mainly with breast and ovarian cancer development. In recent years, germline alterations in these genes have been associated with PCa, and variants in BRCA1 and BRCA2 increase the relative risk of PCa by 3.8 and 8.6 times in men under 65 years of age, respectively. BRCA gene mutations can alter the ability of cells to repair DNA double-strand breaks, contributing to the accumulation of genetic abnormalities that can lead to tumor cell formation [25, 28, 32, 33].

On the other hand, HOX genes contribute to embryonic tissue development, organogenesis, and the regulation of AR target genes. In addition to these functions, HOX genes are involved in cell proliferation, cell cycle, apoptosis, cell differentiation, and migration. For this reason, their mutations can alter the development of various biological processes, increasing the probability of developing PCa. An example is the mutation that generates a substitution of the amino acid glycine for glutamic acid at position 84 of the HOXB13 protein, which is related to the early development of PCa. It is currently known that most mutations that generate alterations in the HOXB13 protein are associated with a risk of developing aggressive PCa in earlier stages of the individual [24, 25, 32–34].

## Age, ethnicity and diet

PCa occurs more frequently as men age, with the average age of diagnosis being 66 years. More than 70% of men older than 80 have histologic changes reflecting PCa [35, 36]. Significantly, the development of PCa at younger ages is directly related to the tumor’s aggressiveness. Black men are more frequently diagnosed with this disease, and its development may even occur at an earlier age in contrast to white and Hispanic men. This may be influenced by genetic factors, such as 5-α-reductase enzyme expression levels, which may be found at higher levels in black men and increase the likelihood of PCa [37, 38]. Another risk factor is diet; it has been observed that a high intake of saturated animal fats, present in red meat and dairy products, is associated with an increased risk of PCa. Some studies suggest that processed meat cooked at high temperatures may increase the production of androgens and increase the levels of reactive oxygen species (ROS), prostaglandins, and leukotrienes, promoting the growth of prostate cancer cells [23, 39–41].

Therefore, early detection of PCa is crucial due to the high incidence of risk factors associated with this disease. This highlights the importance of developing accurate and accessible screening strategies. PCa staging using techniques such as the Gleason scale and imaging allows for personalized and effective patient care, improving long-term outcomes.

## Detection and classification of PCa

PCa can be diagnosed by a digital rectal examination (DRE) or a blood test to measure the plasma PSA concentration [42]. DRE consists of examining the size of the prostate gland and identifying by touch any abnormality, such as lumps or enlargements, that may require a prostate biopsy. DRE is very useful for identifying abnormalities. The PSA test is the main biological test used to identify PCa. PSA is a glycoprotein produced in the prostate gland, and its biological activity includes liquefying seminal serum, contributing to sperm motility, and dissolving cervical mucus [43, 44].

A PSA concentration greater than 4 ng/mL can be a determining factor for performing more tests to confirm or rule out the presence of PCa. Prostate tissue biopsies can be analyzed under a microscope to confirm the presence of cancer cells. Tissue can be removed through the skin between the anus and scrotum or through the rectal wall. These exams are complemented with different imaging techniques, such as multiparametric magnetic resonance imaging (mpMRI), which allows prostate tissue to be visualized using magnetic fields and radio waves. Another method is transrectal ultrasound (TRUS), which allows the diagnosis of possible tumors that cannot be detected by DRE. TRUS involves inserting a transducer that emits sound waves that pass through the prostate and produce echoes that a computer converts to detailed images of different prostate parts [24, 43, 45]. PCa classification is necessary for establishing an adequate treatment regimen. The following provides descriptions of commonly used systems.

The American Joint Committee on Cancer (AJCC) TNM classification is based on the tumor (T), lymph node (N), and metastasis (M) categories. T (primary tumor) is used to classify the tumor’s size and extent, and the T value can vary from T1 to T4. T1 represents a tumor that cannot be evaluated by physical examination or imaging techniques, whereas T4 represents a tumor at a more advanced stage, i.e., the tumor has spread to tissues near the prostate. N (lymph nodes) is used to classify the spread of cancer to lymph nodes, and M (metastasis) denotes the spread of prostate cancer to distant tissues. The values for N and M can be 0 or 1. N0 indicates the absence of spread to the lymph nodes, whereas M0 indicates the absence of spread to other body organs. N1 indicates spread to the lymph nodes, and M1 indicates spread to other body parts [42, 46–49]

The Gleason scale is based on the microscopic appearance of PCa on biopsy, with classification based on the aggressiveness and differentiation of cancer cells [42, 45]. This scale describes histological patterns from 1 to 5, where 1 is a well-differentiated tumor, and 5 is a poorly differentiated tumor with a loss of glandular structure. Tumors receive two grades according to a comparison of the glandular architecture of the tumor cells with that of healthy prostate cells. The primary grade is the pattern most commonly detected in the neoplasm, and the secondary grade is the second most common. Once the pathologist has assigned the primary and secondary scores, they are added to obtain a global score on the Gleason scale; therefore, the global score varies from 2 to 10. Tumors with scores between 2 and 6 are considered low grade or well differentiated, and tumors with 7 to 10 are considered high grade and poorly differentiated [42, 45, 50, 51]. These methods allow physicians to select the most appropriate treatment for patients.

### Current treatments

Treatment begins with active surveillance, e.g., PSA tests, DREs, and periodic biopsies, to monitor PCa progression [52]. Surgical treatments may include removal of the prostate and seminal vesicles and direct bladder neck anastomosis to the urethra [43]. Orchiectomy, another surgical treatment option, involves the removal of the testes, to eliminate the production of androgens and prevent tumor development [43, 53]; however, androgen-depleting drugs, such as luteinizing hormone-releasing hormone agonists or intermittent androgen suppression therapy, are alternative treatments that can be considered [33, 54]. When PCa is limited to the prostate and PSA levels are less than 15 ng/mL, radiation therapy may be considered concomitant with other treatments [21, 43]. In cases where patients are unsuitable for surgery and more targeted irradiation is needed, brachytherapy can be used [21, 55]. When hormonal and surgical treatments have limited effects, one of the most commonly used drugs is docetaxel, which can be combined with steroids such as dexamethasone and has a mechanism of action that alters the depolymerization/polymerization dynamics of the cytoskeleton, leading to the inhibition of cell trafficking and apoptosis [21, 55, 57, 58]. Other treatment regimens are based on drugs such as cabazitaxel combined with steroids, mitoxantrone, estramustine, and carboplatin; however, their use is less common and depends on the response of the patient to other treatments [56].

Because PCa treatments do not exclusively affect tumor cells, side effects tend to occur, such as loss of libido, erectile dysfunction, loss of bone mass, fractures, metabolic syndrome, and cardiovascular morbidity. In addition, sequelae such as emotional and physical disorders, e.g., depression, anxiety, weakness, and general malaise, can occur [21, 59, 60].

The importance of developing other therapies and medications for PCa lies in improving patients’ quality of life and avoiding attrition to treatment owing to sequelae. Research has been carried out to address this need using the components of different snake venoms as potential therapeutic agents.

## Snake venoms

Snake venoms have evolved over millions of years to serve various functions, such as immobilizing prey and self-defense against predators. These venoms are a complex mixture of proteins, peptides, enzymes, and other biochemical components, which can have devastating effects on inoculated organisms [62, 63]. However, in recent decades, scientific research has revealed that these venoms contain compounds with potential therapeutic applications, especially in the fight against cancer [61, 62]. For this reason, there is an increasing interest in the evaluation of molecules with pharmacological activities against cancer cells, such as metalloproteinases, vascular growth factors, phospholipases A_2_ (PLA_2_), cysteine-rich secretory proteins, L-amino acid oxidases, small peptides, C-type lectins, and disintegrins. These molecules have demonstrated the ability to inhibit cell proliferation, induce apoptosis, and suppress angiogenesis [9, 11, 64, 65].

### Snake venoms and their effects on PCa cells

Numerous studies have contributed to understanding the biological mechanisms that explain the antitumor effects of snake venoms on different PCa cell lines (Table 1). Chong et al. (2020) explored the anticancer effects of two poisons from the Asian snakes *Naja sumatrana* and *Naja kaouthia* on the androgen-independent human prostate cancer line PC-3 and reported that complete venom inhibited cell growth in a concentration-dependent manner and had moderate cytotoxicity [66]. A similar cytotoxic effect was observed using the venom of *Montivipera raddei;* a significant reduction in the percentage of viable cells was observed as the concentration of crude venom increased, resulting in 50% PC-3 cell death at a venom concentration of 2.78 µg/mL (IC_50_) [67].

**Table 1:** Effects of snake venoms on prostate cancer cells

Snake	Concentration/Dose	Cell lines	Main effects on Prostate cancer cells	Reference
*Naja sumatrana*	5.58 µg/mL	PC-3	Concentration-dependent cytotoxic activity	[66]
*Naja kaouthia*	9.88 µg/mL			
*Montivipera raddei*	2.78 µg/mL	PC-3		[67]
*Trimeresurus purpureomaculatus*	1.60 µg/mL	PC-3	Cytotoxic activity and cell growth inhibition	[68]
*Montivipera xanthina*	20.0 µg/mL	LNCaP	Concentration- and time-dependent cytotoxicity	[69]
*Naja haje*	38.0 µg/mL	DU-145	Concentration- and time-dependent cytotoxicity	[70]
	42.0 µg/mL	PC-3		
	> 100 µg/mL	LNCaP	Morphological changes	
	61.0 µg/mL	TSUpr1		
*Naja haje*	0.06 µg/mL	PC-3	Concentration-dependent cytotoxicity	[71]
			Upregulation of Bax and downregulation of BCL-2	
			Reduction in PSA and PCA3 expression	
			Cell cycle arrest in the G0-G1 phase	
*Cerastes cerastes*	3.21 µg/mL	PC-3	Reduction of cancer cell viability by individual action and in combination with *Androctonus amoreuxi* venom.	[72]
			Decreased BCL-2 expression	
			Increased Bax/BCL-2 ratio and oxidative stress	
			Cytotoxicity and antiproliferative effect on cells	
*Cerastes cerastes*	18 µg/mL (IRRV) 40 µg/mL (CV) 119 µg/mL (CV-PEE)	PC-3	Complete venom (CV) and irradiated venom (IRRV) generated concentration-dependent toxicity	[73]
			Upregulation of proapoptotic genes such as P53 and Casp-3	
			Downregulation of BCL-2 antiapoptotic gene	
			Alterations in the cell cycle due to the accumulation of DNA in the G2/M phase by treatment with CV and IRRV	
			Oxidative stress in cells treated with CV, IRRV and whole venom mixed with ethanolic propolis (CV-PEE) by ROS generation	
*Walterinnesia aegyptia*	10.0 µg/mL (WEV) 5.0 µg/mL (WEV + NP)	PC-3 LNCaP	WEV and WEV + NP shows concentration- and time-dependent cytotoxicity	[74]
			WEV and WEV + NP reduced the chemokine receptors expression on PCa cell lines	
			WEV + NP affected migration, invasion and proliferation	
			WEV and WEV + NP generated changes in the levels of BCL-2 family proteins	
			WEV and WEV + NP induced apoptosis	
*Walterinnesia aegyptia*	2,5 µg/kg/día (WEV) 1 µg/kg/día (WEV + NP)	PC-3	WEV and WEV + NP inhibited tumor growth in BALB/c murine models	[75]
			WEV + NP and WEV increased oxidative stress levels and reduced chemokine levels	
			WEV and WEV + NP induced apoptosis	
			WEV and WEV + NP inhibited the cellular proliferation	
			WEV and WEV + NP decreased the VEGF levels	
			WEV and WEV + NP decreased the membrane potential	
*Montivipera bulgardaghica albizona*	0,44 µg/mL	DU-145	Concentration-dependent cytotoxicity	[76]
			Apoptosis induction	
	1,51 µg/mL	PC-3	Increase in pro-apoptotic proteins and genes	
			Decrease in anti-apoptotic proteins and genes	

The venom of *Trimeresurus purpureomaculatus*, native to Malaysia, exhibited greater cytotoxicity toward PC-3 prostate cancer cells than RWPE-1 cells, a cell line derived from normal prostate tissue. In addition, venom had a greater capacity to inhibit cancer cell growth than 5-fluorouracil, a widely used drug to treat various types of cancer [68]. The anticancer potential of the venom of *Montivipera xanthina* was also evaluated using the carcinogenic line LNCaP, which is a castration-sensitive cell line, and exhibited concentration-dependent and exposure time-dependent cytotoxicity, as evidenced by changes in morphology and the inhibition of cellular growth; evaluations of the noncancer Vero cell line, which is derived from African green monkey kidney epithelial cells, did not reveal significant cytotoxic effects [69]. Research has also been conducted to evaluate the effects of *Naja haje* snake venom on cancer cell lines such as TSUpr1, derived from human bladder carcinoma, DU-145, and PC-3, both of which are castration-resistant prostate cancer cell lines, and LNCaP.

The toxic effect of *Naja haje* venom on these malignant prostate cells was dependent on the concentration and duration of exposure. Morphological changes in the cells were observed, such as fragmentation and the appearance of vacuoles between adherent cells, ultimately resulting in swelling and cell death. The biomolecules that compose venom may generate lesions that lead to the activation of a cell death program that generates necrotic effects and irreparable damage in treated cells [70]. In another study, Elrefay et al. (2019) reported that the venom of *Naja haje* reduced the viability of PC-3 cells in a concentration-dependent manner. Treatment with the venom also resulted in the overexpression of the proapoptotic gene Bax and the suppressed expression of the antiapoptotic gene BCL-2. In addition, venom reduced the expression of specific PCa biomarkers, such as PSA and PCA3. Furthermore, the venom arrested PC-3 cells in the G0/G1 phase, preventing the initiation of DNA synthesis and cancer cells from continuing the cell replication process [71].

In 2017, the antiproliferative effects of the venom from *Cerastes cerastes* combined with the venom from the scorpion *Androctonus amoreuxiscorpion* were investigated, revealing that both venoms generated a concentration-dependent reduction in the viability of PC-3 cancer cells. A mixture of these venoms decreased the expression of BCL-2, a protein involved in inhibiting apoptosis. In addition, there was an increase in the Bax/BCL-2 protein ratio, promoting programmed cell death. High lipid peroxidation levels were also observed, indicating that venoms may increase oxidative stress in PC-3 cells [72].

Abdelglil et al. (2021) conducted various tests with the venom of the snake *Cerastes cerastes* in combination with ethanolic propolis (CV-PEE) and γ-irradiated venom with ^60^Co (IRRV). While CV-PEE had an effect similar to venom alone, IRRV treatment had the greatest effect on the viability of PC-3 cells in a concentration-dependent manner, with the greatest increase in the levels of proapoptotic proteins. IRRV deregulated the cell cycle, downregulated the expression of the antiapoptotic gene BCL-2, and increased oxidative stress [73].

*Walterinnesia aegyptia* venom (WEV) loaded in silica nanoparticles (NPs) was evaluated by Badr et al. (2013) in the PC-3 and LNCaP cell lines and PCa cells obtained from human tissues. The evaluation of WEV and WEV + NP cytotoxicity on PC-3 cells and primary cells demonstrated a direct relationship between the concentration of venom, the exposure time, and decreases in cell viability; notably, treatment with NPs alone had no cytotoxic effect. In the same study, the regulation of the expression of chemokine receptors in cell lines was also analyzed, and the results indicated a possible suppressive effect on the expression of specific chemokine receptors in PCa lines that are involved in promoting cell invasion and metastasis. In addition, a decrease in the ability of cancer cells to migrate and invade tissues and in EGF-induced proliferation was observed. Cell cycle alterations induced by WEV and WEV + NPs were also observed, with an increase in the number of apoptotic cells, a decrease in cell cycle progression, and the expression of antiapoptotic proteins such as BCL-2 and BCL-XL and a concurrent increase in the expression of proapoptotic proteins such as Bak and Bax [74].

The same authors developed studies with WEV and WEV + NPs using prostate cancer cell line-derived xenograft BALB/c models and reported a decrease in tumor volume and an increase in free radical levels, which possibly generated an increase in the activity of apoptotic caspases 3, 8, and 9. There was also an increase in the oxidative stress level and a reduction in chemokine levels, indicating an inhibitory effect on tumor spread. The treatment also decreased the vascular endothelial growth factor (VEGF) expression, which is important in forming new blood vessels that fuel tumor growth. Thus, WEV and its combination with NPs interfered with tumor vascularization, limiting the ability of tumors to obtain nutrients and oxygen and thus affecting tumor growth. Additionally, treatment with WEV or WEV + NPs significantly reduced the proliferation induced by IGF-1 and EGF in prostate cancer cells. Treatment with WEV alone or in conjunction with NPs induced PCa cell apoptosis, an effect related to increased caspase 3, 8, and 9 activity and decreased antiapoptotic gene expression. Additionally, WEV + NPs decreased the phosphorylation of proteins that contribute to cell survival, such as AKT and ERK, and contributed to the destabilization of the mitochondrial membrane potential of cancer cells [75].

Evaluations of the effects of the venom of *Montivipera bulgardaghica albizona* on the DU-145 and PC-3 tumor lines, and the nontumorigenic line HEK-293 demonstrated that the cytotoxic activity was dependent on the concentration of the venom, with greater selectivity toward PC-3 cells. The venom decreased the percentage of viable cells and increased the proportion of cells in both cancer cell lines’ early and late stages of apoptosis. Additionally, the levels of proteins related to apoptosis were altered in both cell lines before venom treatment. In PC-3 cells, the expression of proapoptotic proteins such as Bad, cleaved caspase-3, catalase, TRAIL R1/DR4, TRAIL R2/DR5, and phospho-p53 significantly increased, whereas the expression of antiapoptotic proteins such as BCL-xL, XIAP, and survivin decreased. In DU-145 cells, the expression of the Bax, cleaved caspase-3, TRAIL R1/DR4, TRAIL R2/DR5, and FAS proteins increased, whereas that of the BCL-2 and CIAP-1 proteins decreased. An analysis of gene expression in PC-3 cells treated with venom revealed that the expression of genes encoding Bad, caspase-3, catalase, TRAIL R1/DR4, TRAIL R2/DR5, and phospho-p53 increased significantly, whereas the expression of the gene encoding XIAP decreased. In DU-145 cells treated with venom, the expression of the genes encoding Bax, caspase-3, TRAIL R1/DR4, TRAIL R2/DR5 and phospho-p53 increased, whereas the expression of the genes encoding BCL-2 and CIAP-1 decreased. These results indicate that venom affects the levels of the main components of the intrinsic and extrinsic apoptotic pathways [76].

These investigations show that venoms could have therapeutic effects on PCa and that the combined use of supplements and venom can potentiate the pharmacological properties of the venom. However, the purification of the individual venom components is essential to understand how they act on tumor cells and evaluate their safety and efficacy. In addition, purification allows for identifying and characterizing specific proteins that can have completely different effects than complete venom and reduce damage to nontumor cells.

### Antitumor effects of snake venom peptides on prostate cancer cells

Snake venom peptides represent a unique class of bioactive compounds that can affect multiple aspects of cancer development and progression. Their ability to induce the selective death of cancer cells, inhibit angiogenesis, and modulate key signaling pathways offers significant therapeutic potential in the fight against PCa [7, 10, 77]. Peptides isolated from snake venoms have been shown to induce selective cytotoxicity in cancer cells without significantly affecting nontumor cells. Some snake venom peptides can induce the apoptosis of cancer cells and inhibit the formation of new blood vessels (angiogenesis) essential for tumor growth [8, 77, 78]. Studies evaluating the effects of snake venom peptides on PCa cells are summarized in Table 2.

**Table 2:** Effect of snake venom peptides on prostate cancer cells

Snake	Peptide	Concentration (µg/mL)	Cell lines	Main effects on prostate cancer cells	Reference
*Vipera lebetina turanica*	SVT	1.7	PC-3	Inhibition of cancer cell line growth	[79]
				Induced cell cycle arrest	
		1.8	DU-145		
				Increased expression of pro-apoptotic proteins	
		9.1	LNCaP		
				Downregulation of anti-apoptotic proteins	
*Trimeresurus gramineus*	Trigramin	1.2	PC-3	Prevention of TCIPA (PC-3 cell-induced platelet aggregation)	[80]
*Calloselasma rhodostoma*	Rhodostomin	0.5			
*Crotalus durissus terrificus*	Crotamine	2.5–10.0	DU-145	Cell migration inhibition	[81]

SVT, a peptide isolated from the venom of *Vipera lebetina turanica*, was evaluated in the PC-3, DU-145, and LNCaP cell lines. This toxin inhibited the growth of the androgen-independent cell lines DU-145 and PC-3, with less of an effect on the growth of LNCaP cells. The treatment generated morphological changes in the cells, such as cytoplasmic blebbing, cell contraction, and cytoplasmic condensation, characteristic of apoptosis. Furthermore, TUNEL assays revealed an increase in apoptotic cells in response to SVT. The treated cells presented low DNA binding activity to nuclear factor-kB, an antiapoptotic factor. Furthermore, SVT increased the expression of proapoptotic genes such as Bax, Caspase 3, Caspase 9, and p53 and negatively regulated the expression of the antiapoptotic gene BCL-2, promoting the activation of programmed cell death pathways in cancer cells. SVT also caused cell cycle arrest in the G0‒G1 and G2‒M phases in PC-3 cells, affecting cell growth [79].

The peptides trigramine and rhodostomina were isolated from the venoms of *Trimeresurus gramineus* and *Calloselasma rhodostoma*, respectively, and their effects on the PC-3 tumor line were evaluated. Both peptides prevented tumor cell-induced platelet aggregation and blocked the interaction of fibrinogen with its specific receptor associated with the glycoprotein IIb-IIIa complex on platelets. Such blockage can interfere with the final common pathway of platelet aggregation induced by various stimuli, such as ADP, epinephrine, thrombin, and other factors, decreasing the ability of malignant cells to cause platelet aggregation, making it difficult for cancer cells to spread through the bloodstream. Thus, these peptides reduced the ability of tumor cells to travel and establish themselves in other body tissues [80].

Crotamine, a peptide isolated from the venom of the snake *Crotalus durissus terrificus*, inhibited the migration of DU-145 cells without affecting their viability. The peptide did not cause cytotoxicity or affect the migration of nontumorigenic human umbilical vein endothelial cells (HUVECs) [81]. Although more research and development are needed, snake venom peptides can potentially become valuable tools in the arsenal of PCa treatments in the future.

## Effects of proteins isolated from snake venoms on prostate cancer cells

### L-amino acid oxidases

L-Amino acid oxidases (LAAOs) are enzymes that specifically oxidize L-amino acids in peptides and proteins, producing hydrogen peroxide (H_2_O_2_) and other ROS, which can induce oxidative stress in cancer cells. These molecules’ unique biochemical properties make them valuable in cancer research [7]. The pharmacological potential of LAAOs lies in their participation in platelet aggregation processes, hemolytic or hemorrhagic effects, cytotoxicity, and apoptosis [82, 83]. Oxidative stress caused by LAAOs can activate intracellular signaling pathways that lead to apoptosis in abnormal cells. OH-LAAO, from the venom of *Ophiophagus hannah*, has shown antiproliferative activity in tumorigenic cells; its cytotoxic activity in the PC-3 line was concentration-dependent and reduced viability at relatively low concentrations; in addition, it increased caspase 3 and 7 activity, indicating that the cell death mediated by *OH-LAAO* in PC-3 cells occurred through apoptosis. Furthermore, PE-annexin V/7-AAD analysis revealed that populations of apoptotic cells were greater in the group treated with *OH-LAAO* than in the control group. In prostate cancer cell line (PC-3)-derived xenograft models, *OH-LAAO* altered the tumor growth rate and caused complete tumor regression in one of the xenograft models without causing pathological abnormalities in the measured biomarkers. That was the first study to demonstrate the antitumor effect of LAAO from the venom of *Ophiophagus hannah* on in vitro and in vivo models of prostate cancer [84].

The LAAO of the venom of *Bothrops moojeni*, known as *Pollonein-LAAO*, was evaluated in the PC-3 cancer cell line. The results indicated that alterations in the viability of malignant cells were associated with an increase in the production of ROS, particularly H_2_O_2_. This effect was blocked by inhibiting LAAO with the enzyme catalase, an antioxidant that breaks down H_2_O_2_ into water and oxygen. Treatment of the PC-3 cell line with *Pollonein*-*LAAO* induced apoptosis in a concentration-dependent manner, with increases in the expression of the TP53, BAX, and BAD genes and a decrease in the expression of the antiapoptotic gene BCL-2. That study further demonstrated that treatment with *Pollonein-LAAO* activated the extrinsic apoptosis pathway in addition to the intrinsic apoptosis pathway by increasing the expression of the cell death receptor DR5, which in turn activated caspase 8, a key enzyme in the cascade of events leading to programmed cell death.

Another important effect of *Pollonein-LAAO* is regulating the expression of genes involved in the transition from the G1 phase to the S phase of the cell cycle. This includes the activation of CDKN1A, which encodes the protein p21, an inhibitor of cyclin-dependent kinases that block cell cycle progression, contributing to inhibiting tumorigenic cell growth. In addition, the effect of LAAO on PC-3 multicellular tumor spheroids (MCTS) was evaluated, as these models are closer to the complexity of the tumor environment than are two-dimensional cell cultures. LAAO affected cell migration, adhesion, and invasion by reducing the ability of PC-3 cells to adhere to substrates of the extracellular matrix, such as type IV collagen, fibronectin, and Matrigel, an effect that was related to alterations in the expression of integrins. These findings indicate that *Pollonein-LAAO* significantly affects multiple aspects of PCa cell biology, demonstrating its high therapeutic potential as an anticancer agent [85].

Another LAAO, *Cmp-LAAO*, from the venom of *Crotalus mitchellii pyrrhus* was shown to generate oxidative stress in the LNCaP cell line. In addition, this enzyme had a concentration-dependent and time-dependent cytotoxic effect on LNCaP cells, with the IC_50_ being lower for cancer cells than for normal RWPE-1 prostate epithelial cells. In addition, *Cmp-LAAO* induced LNCaP cell apoptosis, as evidenced by the externalization of phosphatidylserine in the plasma membrane and the increase in caspase 3 and caspase 9 activities. Alterations in the mitochondrial membrane potential of the cells were also detected. LNCaP cells treated with *Cmp-LAAO* exhibited changes in mitochondrial function. In that study, cotreatment with catalases significantly reduced the cytotoxic effect of *Cmp-LAAO*. These results support the hypothesis that ROS formation is a key event triggering the cytotoxic effect of *Cmp-LAAO* [86]. Similar results to those described above were observed with another LAAO derived from the venom of the *Bungarus multicinctus*: BM-Apotxin. This enzyme generated concentration-dependent cytotoxicity in PC-3 cells. After 24 h of incubation, a growth inhibition rate of 80% was observed, an effect determined to be related to late apoptosis. When cotreated with catalase, both the growth inhibition rate and the percentage of apoptotic cells decreased, which suggests that the cytotoxicity of BM-Apotxin is also related to the generation of H_2_O_2_ as a product of the oxidation of amino acids [87]. These results demonstrate that LAAOs have significant potential in developing drug prototypes against PCa.

### Metalloproteinases

Metalloproteinases can degrade extracellular matrix components, such as collagen and elastin. Metalloproteinases can influence the immune response and angiogenesis within the tumor microenvironment, impacting cancer progression [7]. VLAIP, a metalloproteinase from the venom of *Vipera lebetina turanica*, was evaluated in the PC-3 and LNCaP cell lines. Following VLAIP treatment, a concentration-dependent decrease in cell viability was observed after 24 h of exposure. In addition, VLAIP altered cell morphology and caused the shedding of PC-3 cells. VLAIP also inhibited the ability of PC-3 cells to adhere to the extracellular matrix proteins collagen, fibronectin, and vitronectin. In contrast, LNCaP cells showed a different response; their viability was not significantly affected by treatment, even at concentrations similar to those used for PC-3 cells. These findings suggest that VLAIP has selectivity for different prostate cancer cell lines. These results demonstrate the potential of VLAIP as a possible therapeutic agent against PCa; however, it is necessary to characterize the cytotoxic mechanisms, explore the clinical applicability, and understand the reasons for different effects on different PCa cell lines [88].

### Phospholipases A_2_

PLA_2s_ are found in different animal venoms, such as bees, scorpions, and snakes, where they play a catalytic role in the hydrolysis of phospholipids at the sn-2 position of the ester bond, allowing the release of fatty acids and lysophospholipids [89]. PLA_2_ has toxic effects on cancer cells, with moderate toxicity to nontumor cells, providing a basis for new research focused on developing cancer treatments [7, 90]. A recent study investigated the effects of *Bothropstoxin-I* (*BthTX-I*), a PLA_2_ isolated from the venom of *Bothrops jararacussu.* A resazurin reduction assay revealed that *BthTX-I* significantly decreased the viability of HUVECs and DU-145 cells. A comet assay performed with DU-145 cells treated with *BthTX-I* also revealed the concentration-dependent generation of DNA damage. These findings suggest that exposure to PLA_2_ induces genetic damage in PCa cells, which could affect cancer progression. However, the protein also affects nontumorigenic HUVECs, which makes it necessary to perform investigations focused on determining its effect on healthy tissues [91].

Another study with svPLA_2_ isolated from the venom of *Naja haje* revealed a concentration-dependent cytotoxic effect on PC-3 cells. Treatment with SvPLA_2_ resulted in the overexpression of the proapoptotic gene Bax and the suppressed expression of the antiapoptotic gene BCL-2 in PC-3 cells. In addition, it reduced the expression of specific PCa biomarkers, such as PSA and PCA3. Finally, a cell cycle analysis revealed that treatment with svPLA_2_ resulted in G0/G1 phase arrest [71]. Another svPLA_2_ from the venom of the krait *Bungarus fasciatus* showed a cytotoxic effect on the PC-3 and LNCaP cancer lines, while treatment with PLA_2_ did not decrease the viability of HK-2 cells (a proximal tubular cell line derived from the healthy kidney) [92].

Recently, the *Nutrition Group* (a research group at Universidad del Valle, Cali, Colombia) evaluated the cytotoxic effects of the phospholipase *Pllans-II*, which was isolated from the venom of the snake *Porthidium lansbergii lansbergii*, on the LNCaP, DU-145, and PC-3 PCa cell lines and the nontumorigenic PCS-440-010 prostate cell line. *Pllans-II* had the greatest cytotoxic effects on LNCaP cells, with an approximate 65% reduction in viability, followed by DU-145 cells, with an approximate 45% reduction in viability, and PC-3 cells, with an approximate 28% reduction in viability. *Pllans-II* also inhibited the migration, adhesion, invasion, and proliferation of LNCaP cells. Interestingly, in the nontumorigenic line, *Pllans-II* did not generate greater than 18% cytotoxicity (unpublished data).

### Disintegrins

Disintegrins from snake venoms are potential antitumor agents because they can affect tumor cells’ survival and proliferation pathways. Disintegrins exhibit inhibitory effects on certain integrins and may be important in biomedical research. Integrins that express the recognition sequence to the RGD motif bind to extracellular matrix proteins with the RGD motif, such as vitronectin and fibronectin; disintegrins expressing this motif can affect substrate adhesion to extracellular matrix proteins. The MLD motif is exclusive to heterodimeric disintegrins and can inhibit integrins such as 4β1, 4β7, and 9β1. KTS motif disintegrins are monomeric and selectively bind to the 1β1 collagen receptor [93]. These biomolecules exhibit high specificity and selectivity toward the integrins of cancer cells, affecting cell proliferation, metastasis, angiogenesis, and the modulation of immune responses [78]. Studies of disintegrins can help design drugs for targeted therapy to disrupt the adhesion, migration, and proliferation of cancer cells and reduce the side effects of current treatments [62, 78, 94].

Contortrostatin (CN), a disintegrin isolated from the venom of the snake *Agkistrodon contortrix*, was evaluated alone and in combination with the chemotherapeutic agent docetaxel in the PC-3 and CWR-22 cell lines (androgen-dependent PCa cell lines). That study revealed that CN had a cytotoxic effect, with increased cytotoxicity in combination with docetaxel. CN also inhibited the adhesion of PC-3 cells and, to a lesser extent, that of CWR-22 cells, in particular by altering the binding to fibronectin and, to a lesser extent, vitronectin. Furthermore, disintegrins have been shown to inhibit the migration of PC-3 cells in Matrigel. In vivo, the combination of CN and docetaxel was evaluated in 22 tumors from prostate cancer cell line (PC-3 and CWR-22)-derived xenograft models, with significant synergism observed in the combined treatment compared with the individual treatments, resulting in a decrease in tumor volume compared with that in untreated mice. CN also inhibited angiogenesis induced by basic fibroblast growth factor (bFGF) and vascular endothelial growth factor (VEGF), indicating that CN may interfere with forming new blood vessels in prostate tumors, limiting their growth. Finally, because prostate cancer tends to metastasize to bones, the effect of CN on prostate tumors caused by PC-3 cells implanted directly in mouse bones was evaluated. CN inhibited the growth of these tumors in the bone environment, both individually and in combination with docetaxel, an effect that is relevant for the development of drugs that affect bone metastatic prostate cancer [95].

The recombinant version of CN, i.e., Vicrostatin (VCN), was evaluated in prostate cancer cell line (PC-3)-derived xenograft models after liposomal formulation (LVCN) preparation. LVCN significantly reduced tumor growth in treated mice compared with control and VCN-treated mice; an antiangiogenic effect was also evidenced by a reduction in the density of microvessels in the treated tumors. In addition, a study with LVCN was performed in mouse models with PCa bone metastasis using the CWR22rV1 cell line. In that study, compared with nonliposomal treatment, VCN significantly inhibited the development of bone metastasis. This effect could have been due to improved permeability and retention in the tumor environment due to the encapsulation of VCN in liposomes [96]. Other disintegrins that have been studied include rodostomine, trigramine, and triflavin from the venoms of the snakes *Calloselasma rhodostoma*, *Trimeresurus gramineus*, and *Trimeresurus flavoviridis*, respectively. These disintegrins inhibited the ability of PC-3 cells to adhere to nonmineralized and mineralized bone extracellular matrices and inhibited the migration of cancer cells toward a chemoattractant environment. In addition, disintegrins have been shown to prevent the invasion of cells through Matrigel, which mimics the barriers that tumor cells must overcome during tissue invasion. The identification of binding receptors in tumor cells revealed that Rodostomina specifically blocks the interactions of integrin αvβ3 with tumor cells, which suggests that this interaction is key to the inhibitory effect of Rodostomina on the adhesion and migration of tumor cells. The binding of this integrin with extracellular matrix proteins triggered a series of intracellular events that promoted the adhesion, migration, and invasion of tumor cells [97].

**Fig. 2:**
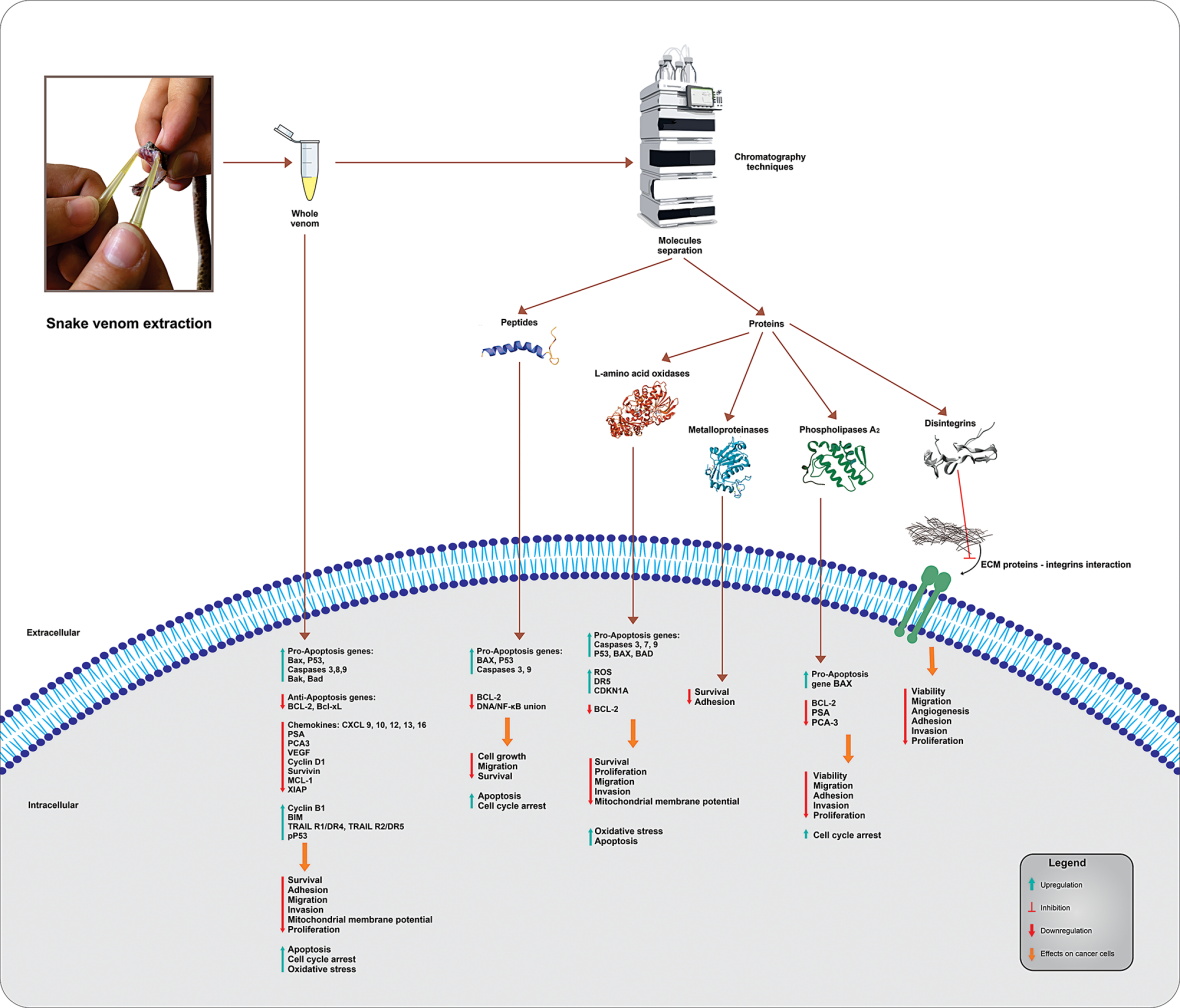
Principal effects of snake venoms and its components on prostate cell cancer

Recently, in the *Nutrition Group* of Universidad del Valle, the cytotoxic effect of *Lansbermin-I*, a disintegrin isolated from the venom of the snake *Porthidium lansbergii lansbergii*, on the PCa, DU-145 and PC-3 tumorigenic cell lines was evaluated. *Lansbermin-I* decreased the viability of DU-145 cells by 40%, whereas for PC-3 cells, the decrease was only 10%. Furthermore, *Lansbermin-I* significantly inhibited the adhesion of DU-145 cells to fibronectin substrates (80% inhibition at a concentration of 50 µg/mL). This finding suggests disintegrins may interfere with α5β1 integrin receptors associated with fibronectin interactions. Interestingly, *Lansbermin-I* inhibited the interaction of cells with Matrigel and collagen to a lesser extent. Furthermore, disintegrins have been shown to inhibit the migration and colony formation ability of DU145 cells (unpublished data). Figure 2 describes the principal effects produced by whole venom, peptides, and proteins from snake venoms against prostate cancer cells.

The results described above suggest that it is worthwhile to continue developing research with disintegrins from snake venoms in the search for molecules that selectively attack PCa cells, as they have the potential to inhibit cell adhesion, migration, and invasion. Table 3 summarizes the cytotoxic and antitumor effects of proteins from snake venom on prostate cancer cells.

**Table 3:** Effect of proteins isolated from snake venom on prostate cancer cells

Snake	Protein	Concentration/Dose	Cell line (s)	Main effects on prostate cancer cells	Reference
*Ophiophagus hannah*	OH-LAAO [L-Amino acid oxidase]	0.05 µg/mL 1 µg/g/day/200 µL	PC-3	Concentration-dependent cytotoxicity	[84]
				Decreased tumor growth in in vivo models	
				No pathological abnormalities in vital organs in murine models	
*Bothrops moojeni*	*Pollonein-*LAAO [L-Amino acid oxidase]	0.09–3.125 µg/mL	PC-3	Decreased cell viability	[85]
				Apoptosis induction	
				Induced changes in the expression of genes related to apoptosis, cell cycle, and cell invasion	
				Cytotoxic effect on MCTS	
				Cell invasion, migration, and adhesion inhibition	
*Crotalus mitchellii pyrrhus*	*Cmp-*LAAO [L-Amino acid oxidase]	0.5–4.0 µg/mL	LNCaP	Concentration- and time-dependent cytotoxicity	[86]
				Apoptosis induction and oxidative stress by ROS generation	
				Selectivity towards cancer cells over the healthy RWPE-1 cell line	
				Membrane potential alteration	
*Bungarus multicinctus*	BM-Apotxin [L-Amino acid oxidase]	113.1 nM	PC-3	Concentration-dependent cytotoxicity and cell growth inhibition	[87]
				Increased percentage of late apoptotic cells	
*Vipera lebetina turanica*	VLAIP [Metalloproteinase]	1.0–100 µg/mL	PC-3 LNCaP	Decreased viability in a concentration- and time-dependent manner on PC-3	[88]
				Morphological changes	
				Impaired cell adhesion to matrix proteins	
				Decreased cell viability	
*Bothrops Jararacussu*	*Bothropstoxin-I* (*BthTX-I*) [Phospholipase A_2_]	1–50 µg/mL	DU-145	Reduced cell viability	[91]
				Genotoxic effects due to the promotion of DNA damage	
*Naja haje*	svPLA_2_	0.84 µg/mL	PC-3	Concentration-dependent cytotoxicity	[71]
				Bax gene overexpression	
				BCl-2 gene underexpression	
				Reduction in PSA and PCA3 expression	
				Cell cycle arrest in the G0/G1 phase	
*krait Bungarus fasciatus*	svPLA_2_	100.0 µg/mL	PC-3 LNCaP HK-2	Cytotoxic effect on PCa cells without affecting the viability of the non-tumorigenic cell line	[92]
*Porthidium lansbergii lansbergii*	*Pllans-II* [Phospholipase A_2_]	100 µg/mL	LNCaP DU-145 PC-3 PCS-440-010	Cytotoxic effect on LNCaP and DU-145 cell lines	Vela-Prieto, C. et al. [No date]
				The viability of non-tumorigenic prostate cells (PCS-440-010) was not inhibited	
				The migration, invasion, and adhesion of LNCaP cell lines were inhibited	
				Colony formation inhibition of LNCaP cells	
*Agkistrodon contortrix contortrix*	Contortrostatin [Disintegrin]	100 nM (CN) 60 µg/day	PC-3 CWR-22	CN inhibited the proliferation and adhesion of prostate cancer cells	[95]
				High cytotoxicity through synergism with Docetaxel	
				CN affected the angiogenesis process in biological models	
				CN inhibited tumor growth in prostate cancer cell line-derived xenograft models	
*Agkistrodon contortrix contortrix*	Vicrostatin (liposomal) [rDisintegrin]	100 µg/dose	PC-3 CWR22rV1	Reduction in the growth of metastatic bone cancer	[96]
				Reduction of tumor growth in in vivo models	
				Decrease in tumor-associated microvessels	
*Calloselasma rhodostoma*	Rodostomine [Disintegrin]	1–10 µg/mL	PC-3	Adhesion inhibition to mineralized bone cell matrices	[97]
				Migration and invasion inhibition of prostate tumor cells	
*Trimeresurus gramineus*	Trigramine [Disintegrin]	10 µg/mL		Adhesion inhibition to nonmineralized and mineralized bone extracellular matrices	
				Cell migration and invasion inhibition processes	
*Trimeresurus flavoviridis*	Triflavin [Disintegrin]	10 µg/mL		Adhesion inhibition to nonmineralized and mineralized bone extracellular matrices	
				Cell migration and invasion inhibition of prostate tumor cells	
*Porthidium lansbergii lansbergii*	*Lansbermin-I* [Disintegrin]	50 µg/mL	PC-3 DU-145	Cytotoxic effect on DU-145	Ochoa-Mosquera, J. et al. [No date]
				Cell migration and adhesion inhibition	
				Colony formation inhibition	

Several investigations have been developed using snake venom and its components in PCa. Among these studies, the action of the disintegrin family stands out, as they have demonstrated significant anticancer activity by inhibiting crucial processes involved in the development and metastasis of PCa. Among these proteins is contortrostatin, one of the most studied disintegrins in the field of bioprospecting focused on oncology. Contortrostatin has shown anticancer effects on breast, ovarian, melanoma, and PCa cell lines, affecting cancer cell proliferation, adhesion, and migration. Its cytotoxic action is mainly attributed to its interaction with extracellular matrix components, such as integrin-type receptors. Likewise, it is essential to recognize the effect of other biomolecules from snake venom on different cancer types, such as phospholipases A2 and L-amino oxidases, which have shown similar results to disintegrins, affecting key processes in the cancer cells’ progression.

### Limitations

Despite these findings, there is a pressing need for further research in this area. Current work is strongly inclined to develop experiments on PCa with whole venom, and studies focusing on the anticancer evaluation of isolated venom components are limited to a few experimental assays, such as cytotoxicity, migration, and adhesion activity. This limitation hinders a better understanding of their molecular mechanisms of action. Additionally, although in vitro studies on PCa cell lines and in vivo studies have demonstrated the anticancer potential of these compounds, their clinical applicability remains uncertain. There is a lack of studies on the safety of these compounds, which is also related to the shortage of preclinical and clinical research, thus slowing the development of these components as potential drugs. Therefore, it is crucial to research the proteins and peptides isolated from snake venom to fully understand these biomolecules’ action mechanisms, optimize their use, and overcome current barriers.

## Conclusions

Prostate cancer is a widespread disease that affects many men globally. This study highlights the potential of using snake venoms and their constituent biomolecules to target prostate cancer. Research has shown that many of the molecules found in snake venoms have various anti-tumor effects, such as inhibiting cell growth, triggering apoptosis, reducing angiogenesis, and interfering with tumor cell adhesion, migration, and invasion. These findings suggest that snake venom components could be a promising area for exploring the treatment of prostate cancer. However, it’s important to note that this research is still in its early stages, and significant effort is needed to understand the underlying mechanisms of the anticancer potential. Challenges include the need for studies specific to prostate cancer and overcoming limitations when translating results from laboratory assays to clinical investigations. Exploring innovative technologies, such as using nanomaterials to improve bioavailability and chemical or recombinant synthesis for producing larger quantities of venom proteins, may open new avenues for future preclinical and clinical studies. These efforts could ultimately lead to more effective and less invasive therapies for prostate cancer patients, improving their quality of life and survival rates.

## Data Availability

No datasets were generated or analysed during the current study.

## References

[1] Sung H, Ferlay J, Siegel RL, Laversanne M, Soerjomataram I, Jemal A, Bray F (2021) Global cancer statistics 2020: GLOBOCAN estimates of incidence and mortality worldwide for 36 cancers in 185 countries. CA Cancer J Clin 71(3):209–249. 10.3322/caac.21660 33538338 10.3322/caac.21660

[2] Bray F, Laversanne M, Sung H, Ferlay J, Siegel RL, Soerjomataram I, Jemal A (2024) Global cancer statistics 2022: GLOBOCAN estimates of incidence and mortality worldwide for 36 cancers in 185 countries. CA Cancer J Clin 74(3):229–263. 10.3322/caac.21834 38572751 10.3322/caac.21834

[3] Ferlay J, Ervik M, Lam F, Colombet M, Mery L, Piñeros M, Znaor A, Soerjomataram I, Bray F (2024) Global Cancer Observatory: Cáncer Tomorrow. Lyon, Francia: International Agency for Research on Cancer. https://gco.iarc.fr/tomorrow/en Accessed 9 March 2024

[4] Ferlay J, Ervik M, Lam F, Colombet M, Mery L, Piñeros M, Znaor A, Soerjomataram I, Bray F (2024) Global Cancer Observatory: Colombia. Lyon, Francia: International Agency for Research on Cancer. https://gco.iarc.who.int/media/globocan/factsheets/populations/170-colombia-fact-sheet.pdf Accessed 9 March 2024

[5] American Cancer Society (2024) Tratamiento del cáncer de próstata. https://www.cancer.org/es/cancer/tipos/cancer-de-prostata/tratamiento.html Accessed 2 August 2024

[6] Barreiro D, Castro F, Castro P et al (2023) Guía para El diagnóstico Y tratamiento del cáncer de próstata. Rev Argent Urol 88(1):26–44

[7] Urra FA, Araya-Maturana R (2022) Putting the brakes on tumorigenesis with snake venom toxins: new molecular insights for cancer drug discovery. Semin Cancer Biol 80:195–204. 10.1016/j.semcancer.2020.05.006 32428714 10.1016/j.semcancer.2020.05.006

[8] Beeton C, Gutman GA, Chandy G (2006) Targets and therapeutic properties of venom peptides. Handb Biologically Act Peptides 2(58):403–413. 10.1016/B978-012369442-3/50061-1

[9] Koh D, Armugam A, Jeyaseelan K (2006) Snake venom components and their applications in biomedicine. Cell Mol Life Sci 63(24):3030–3041. 10.1007/s00018-006-6315-0 17103111 10.1007/s00018-006-6315-0 PMC11135979

[10] Pennington MW, Czerwinski A, Norton RS (2018) Peptide therapeutics from venom: current status and potential. Bioorg Med Chem 26(10):2738–2758. 10.1016/j.bmc.2017.09.029 28988749 10.1016/j.bmc.2017.09.029

[11] Li L, Huang J, Lin Y (2018) Snake venoms in cancer therapy: past, present and future. Toxins 10(9):346. 10.3390/toxins10090346 30158426 10.3390/toxins10090346 PMC6162746

[12] Nosti A (2019) Venenos de serpiente, no tan malos como los pintan. Undergraduate Dissertation, Universidad de Sevilla

[13] Marcinkiewicz C (2013) Applications of snake venom components to modulate integrin activities in cell-matrix interactions. Int J Biochem 45(9):1974–1986. 10.1016/j.biocel.2013.06.009 10.1016/j.biocel.2013.06.009 PMC3774133 23811033

[14] Rebello RJ, Oing C, Knudsen KE, Loeb S, Johnson DC, Reiter RE, Gillessen S, Van der Kwast T, Bristow RG (2021) Prostate cancer. Nat Rev Dis Primers 7(1):9. 10.1038/s41572-020-00243-0 33542230 10.1038/s41572-020-00243-0

[15] Cataño-Cataño JG, Castillo-Londoño JS, Gamboa-Garay OA, Aponte-Varón H, Alvarado-Bestene R, Cortés-Buitrago C (2013) Instituto Nacional de Cancerología. Guía de práctica clínica (GPC) para la detección temprana, seguimiento y rehabilitación del cáncer de próstata. 717:34–83

[16] Haffner MC, Zwart W, Roudier MP, True LD, Nelson WG, Epstein JI, Yegnasubramanian S (2021) Genomic and phenotypic heterogeneity in prostate cancer. Nat Rev Urol 18(2):79–92. 10.1038/s41585-020-00400-w 33328650 10.1038/s41585-020-00400-w PMC7969494

[17] Yanes-Alison VN, Cubas S (2023) Cáncer De próstata: una perspectiva global. Rev Med Sinerg 8(12):1–12. 10.31434/rms.v8i12.1124

[18] National Cancer Institute (2023) Prostate Cancer Treatment (PDQ^®^)–Patient Version. https://www.cancer.gov/types/prostate/patient/prostate-treatment-pdq Accessed 13 March 2024

[19] Muniyan S, Chaturvedi NK, Dwyer JG, Lagrange CA, Chaney WG, Lin MF (2013) Human prostatic acid phosphatase: structure, function and regulation. Int J Mol Sci 14(5):10438–10464. 10.3390/ijms140510438 23698773 10.3390/ijms140510438 PMC3676848

[20] Thomas LN, Lazier CB, Gupta R, Norman RW, Troyer DA, O’brien SP, Rittmaster RS (2005) Differential alterations in 5a-Reductase type 1 and type 2 levels during development and progression of prostate Cancer. Prostate 63:231–239. 10.1002/pros.20188 15538746 10.1002/pros.20188

[21] Blanco A, Escudero de los Ríos PM, Hernández Toríz N (2008) Cáncer De próstata. Rev Mex Urol 68(4):250–259

[22] Pesce G (2011) Cellular interactions that regulate prostate cancer growth, survival and tumourigenicity. Doctoral Dissertation, University of Sheffield. 10.13140/RG.2.2.26238.54088

[23] Aronson WJ, Barnard RJ, Freedland SJ, Henning S, Elashoff D, Jardack PM, Cohen P, Heber D, Kobayashi N (2010) Growth inhibitory effect of low fat diet on prostate cancer cells: results of a prospective, randomized dietary intervention trial in men with prostate cancer. J Urol 183(1):345–350. 10.1016/j.juro.2009.08.104 19914662 10.1016/j.juro.2009.08.104 PMC3089950

[24] Sekhoacha M, Riet K, Motloung P, Gumenku L, Adegoke A, Mashele S (2022) Prostate cancer review: Genetics, diagnosis, treatment options, and alternative approaches. Molecules 27(17):5730. 10.3390/molecules27175730 36080493 10.3390/molecules27175730 PMC9457814

[25] Castro-Mujica M (2022) Clinical implications of the molecular biology of prostate cáncer: review article. Rev Fac Med Hum 22(3):597–613. 10.25176/RFMH.v22i3.5043

[26] Steinestel J, Luedeke M, Arndt A, Schnoeller TJ, Lennerz JK, Wurm C, Maier C, Cronauer MV, Steinestel K, Schrader AJ (2019) Detecting predictive androgen receptor modifications in circulating prostate cancer cells. Oncotarget 10(41):4213–4223. 10.18632/oncotarget.3925 31289619 10.18632/oncotarget.3925 PMC6609250

[27] Shiota M, Akamatsu S, Tsukahara S, Nagakawa S, Matsumoto T, Eto M (2022) Androgen receptor mutations for precision medicine in prostate cancer. Endocr Relat Cancer 29(10):R143–R155. 10.1530/ERC-22-0140 35900853 10.1530/ERC-22-0140

[28] Wang G, Zhao D, Spring D, DePinho R (2018) Genetics and biology of prostate cancer. Genes Dev 32:1105–1140. 10.1101/gad.315739.118 30181359 10.1101/gad.315739.118 PMC6120714

[29] Antonarakis ES, Lu C, Wang H, Luber B, Nakazawa M, Roeser JC et al (2014) AR-V7 and Resistance to Enzalutamide and Abiraterone in prostate Cancer. N Engl J Med 371(11):1028–1038. 10.1056/nejmoa1315815 25184630 10.1056/NEJMoa1315815 PMC4201502

[30] Gholamreza P, Abed-Ali Z, Amir A, Abdolrasoul M, Hossein A, Ali A, Hamid S (2007) Role of PTEN Gene in progression of prostate Cancer. Urol J 4(2):95–100. 10.22037/uj.v4i2.138 17701929

[31] Jamaspishvili T, Berman D, Ross A, Scher H, De Marzo A, Squire J, Lotan T (2018) Clinical implications of PTEN loss in prostate cancer. Nat Rev Urol 15:222–234. 10.1038/nrurol.2018.9 29460925 10.1038/nrurol.2018.9 PMC7472658

[32] Zhen J, Syed J, Anh K, Leapman M, Agarwal N, Brierley K, Llor X, Hofstatter E, Shuch B (2018) Genetic testing for heredetary prostate cáncer: current status and limitations. Cancer 124(15):3105–3117. 10.1002/cncr.31316 29669169 10.1002/cncr.31316

[33] Vietri M, D’elia G, Caliendo G, Resse M, Casamassimi A, Passariello L, Albanese L, Cioffi M, Molinari A (2021) Hereditary prostate Cancer: genes related, Target Therapy and Prevention. Int J Mol Sci 22(7):3753. 10.3390/ijms22073753 33916521 10.3390/ijms22073753 PMC8038462

[34] Brechka H, Bhanvadia R, VanOpstall C, Vander D (2017) HOXB13 mutations and binding partners in prostate development and cancer: function, clinical significance, and future directions. Genes Dis 4(2):75–87. 10.1016/j.gendis.2017.01.003 28798948 10.1016/j.gendis.2017.01.003 PMC5548135

[35] Islas-Pérez LÁ, Martínez-Reséndiz JI, Ruiz-Hernández et al (2020) Epidemiología Del cáncer De próstata, sus determinantes y prevención. J Negat No Posit Results 5(9):1010–1022. 10.19230/jonnpr.3686

[36] Rawla P (2019) Epidemiology of prostate Cancer. World J Oncol 10(2):63–89. 10.14740/wjon1191 31068988 10.14740/wjon1191 PMC6497009

[37] Wang L, Lu B, He M, Wang Y, Wang Z, Du L (2022) Prostate Cancer incidence and mortality: global status and temporal trends in 89 countries from 2000 to 2019. Front Public Health 10:811044. 10.3389/fpubh.2022.811044 35252092 10.3389/fpubh.2022.811044 PMC8888523

[38] Hugosson J, Godtman RA, Carlsson SV, Aus G, Grenabo Bergdahl A, Lodding P et al (2018) Eighteen-year follow-up of the Göteborg Randomized Population-based prostate Cancer screening trial: effect of sociodemographic variables on participation, prostate cancer incidence and mortality. Scand J Urol 52(1):27–37. 10.1080/21681805.2017.1411392 29254399 10.1080/21681805.2017.1411392 PMC5907498

[39] Armstrong B, Doll R (1975) Environmental factors and cancer incidence and mortality in different countries, with special reference to dietary practices. Int J Cancer 15(4):617–631. 10.1002/ijc.2910150411 1140864 10.1002/ijc.2910150411

[40] Venkateswaran V, Klotz LH (2010) Diet and prostate cancer: mechanisms of action and implications for chemoprevention. Nat Rev Urol 7(8):442–453. 10.1038/nrurol.2010.102 20647991 10.1038/nrurol.2010.102

[41] Singh SV, Srivastava SK, Choi S, Lew KL et al (2005) Sulforaphane-induced cell death in human prostate cancer cells is initiated by reactive oxygen species. J Biol Chem 280(20):19911–19924. 10.1074/jbc.M412443200 15764812 10.1074/jbc.M412443200

[42] American Cancer Society (2019) Detección temprana, diagnostico y clasificación por etapas del cáncer de próstata. https://www.cancer.org Accessed 12 April 2024

[43] Ruiz López AI, Pérez Mesa JC, Cruz Batista Y, González Lorenzo LE (2017) Actualización sobre cáncer de próstata. Correo Cient Med 21(3):876–887

[44] Marichal FSF, García RT, Álvarez AV, Roque OS (2015) Cáncer prostático: correlación entre El valor Del antígeno prostático específico y El Resultado anatomoclínico. Rev Arch Med Camagüey 19(1):42–49

[45] Borley N, Feneley M (2009) Prostate cancer: diagnosis and staging. Asian J Androl 11(1):74–80. 10.1038/aja.2008.19 19050692 10.1038/aja.2008.19 PMC3735216

[46] Crawford ED (2009) Understanding the epidemiology, natural history, and key pathways involved in prostate cancer. Urology 73(5):S4. 10.1016/j.urology.2009.03.001 19375626 10.1016/j.urology.2009.03.001

[47] Yin M, Bastacky S, Chandran U, Becich MJ, Dhir R (2008) Prevalence of incidental prostate cancer in the general population: a study of healthy organ donors. J Urol 179(3):892–895. 10.1016/j.juro.2007.10.057 18207193 10.1016/j.juro.2007.10.057

[48] Surasi DSS, Chapin B, Tang C, Ravizzini G, Bathala TK (2020) Imaging and management of prostate cancer. Semin Ultrasound CT MR 41(2):207–221. 10.1053/j.sult.2020.02.001 10.1053/j.sult.2020.02.001 32446432

[49] SchrlSder FH, Hermanek P, Denis L, Fair WR, Gospodarowicz MK (1992) The TNM classification of prostate cancer. Prostate 4:129–138 10.1002/pros.2990210521 1574453

[50] Bolaños P, Chacón C (2017) Escala patológica De Gleason para El cáncer De próstata y sus modificaciones. Med Leg Costa Rica 34(1):237–243

[51] Castillejos-Molina RA, Gabilondo-Navarro FB (2016) Cáncer De próstata. Salud Publ Mex 58(2):279–284 10.21149/spm.v58i2.7797 27557386

[52] Ministerio de Salud de Chile (2015) Guía Clínica auge cáncer de próstata en personas de 15 años y más. https://diprece.minsal.cl/wrdprss_minsal/wp-content/uploads/2016/03/Cancer-de_prostata_25-nov-2015.pdf Accessed 20 May 2024

[53] Jiménez IJ (2021) Tratamiento Del Cáncer De Próstata localizado. NPunto 4(34):4–26

[54] Jaratlerdsiri W, Chan EK, Gong T, Petersen DC et al (2018) Whole-genome sequencing reveals elevated tumor mutational burden and initiating driver mutations in African men with treatment-naïve, high-risk prostate cancer. Cancer Res 78(24):6736–6746. 10.1158/0008-5472.CAN-18-0254 30217929 10.1158/0008-5472.CAN-18-0254

[55] Han Y, Rand KA, Hazelett DJ, Ingles SA et al (2016) Prostate cancer susceptibility in men of African ancestry at 8q24. J Natl Cancer Inst 108(7):djv431. 10.1093/jnci/djv431 26823525 10.1093/jnci/djv431 PMC4948565

[56] American Cancer Society (2024) Treating Prostate Cancer. https://www.cancer.org/cancer/types/prostate-cancer/treating.html Accessed 12 April 2024

[57] Pienta KJ (2001) Preclinical mechanisms of action of docetaxel and docetaxel combinations in prostate cancer. Semin Oncol 28(15):3–7. 10.1016/S0093-7754(01)90148-4 10.1016/s0093-7754(01)90148-4 11685722

[58] Sekino Y, Teishima J (2020) Molecular mechanisms of docetaxel resistance in prostate cancer. Cancer Drug Resist 3(4):676–685. 10.20517/cdr.2020.37 35582222 10.20517/cdr.2020.37 PMC8992564

[59] Morote J, Maldonado X, Morales-Bárrera R (2016) Cáncer De próstata. Med Clin 146(3):121–127. 10.1016/j.medcli.2014.12.021 10.1016/j.medcli.2014.12.021 25727526

[60] American Cancer Society (2023) Tratamiento del cáncer de próstata. https://www.cancer.org/content/dam/CRC/PDF/Public/9000.00.pdf Accessed 13 April 2024

[61] Ma R, Kwok HF (2022) New opportunities and challenges of venom-based and bacteria-derived molecules for anticancer targeted therapy. Semin Cancer Biol 80:356–369. 10.1016/j.semcancer.2020.08.010 32846203 10.1016/j.semcancer.2020.08.010

[62] Mirzaei S, Fekri HS, Hashemi F, Hushmandi K et al (2021) Venom peptides in cancer therapy: an updated review on cellular and molecular aspects. Pharmacol Res 164:105327. 10.1016/j.phrs.2020.105327 33276098 10.1016/j.phrs.2020.105327

[63] Calvete JJ, Juarez P, Sanz L (2007) Snake Venomics Strategy and applications. J Mass Spectrom 42:1405–1414. 10.1002/jms.1242 17621391 10.1002/jms.1242

[64] Montoya-Gómez A, Montealegre-Sánchez L, García-Perdomo H, Jiménez-Charris E (2020) Cervical cancer and potential pharmacological treatment with snake venoms. Mol Biol Rep 47:4709–4721. 10.1007/s11033-020-05503-6 32406018 10.1007/s11033-020-05503-6

[65] Markland F (1998) Snake venoms and the hemostatic system. Toxicon 36:1749–1800. 10.1016/s0041-0101(98)00126-360 9839663 10.1016/s0041-0101(98)00126-3

[66] Chong HP, Tan KY, Tan CH (2020) Cytotoxicity of snake venoms and cytotoxins from two southeast Asian cobras (*Naja sumatrana, Naja kaouthia*): exploration of anticancer potential, selectivity, and cell death mechanism. Front Mol Biosci 7:583587. 10.3389/fmolb.2020.583587 33263003 10.3389/fmolb.2020.583587 PMC7686564

[67] Nalbantsoy A, Hempel BF, Petras D, Heiss P, Göçmen B, Iğci N, Yildiz MZ, Süssmuth RD (2017) Combined venom profiling and cytotoxicity screening of the Radde’s mountain viper (*Montivipera raddei*) and Mount Bulgar Viper (*Montivipera bulgardaghica*) with potent cytotoxicity against human A549 lung carcinoma cells. Toxicon 135:71–83. 10.1016/j.toxicon.2017.06.008 28625888 10.1016/j.toxicon.2017.06.008

[68] Tan CH, Liew JL, Navanesan S, Sim KS, Tan NH, Tan KY (2020) Cytotoxic and anticancer properties of the Malaysian mangrove pit viper (*Trimeresurus purpureomaculatus*) venom and its disintegrin (*purpureomaculin*). J Venom Anim Toxins Incl Trop Dis 26:1–14. 10.1590/1678-9199-jvatitd-2020-0013 10.1590/1678-9199-JVATITD-2020-0013 PMC7375409 32742279

[69] Yalcin HT, Ozen MO, Gocmen B, Nalbantsoy A (2014) Effect of Ottoman viper (*Montivipera xanthina* (Gray, 1849)) venom on various Cancer cells and on microorganisms. Cytotechnology 66:87–94. 10.1007/s10616-013-9540-z 23381026 10.1007/s10616-013-9540-z PMC3886531

[70] Omran MAA (2002) *In vitro* anticancer effect of scorpion *Leiurus quinquestriatus* and Egyptian cobra venom on human breast and prostate cancer cell lines. J Med Sci 3(1):66–86. 10.3923/jms.2003.66.86

[71] Elrefay M, Elfiky A, Sayed R, Zaki H (2019) Snake venom, bee venom and their components exert an anti-cancer effect by triggering apoptosis and cell cycle arrest in prostate cancer. Bull Fac Pharm 57(2):148–156. 10.21608/bfpc.2019.101875

[72] Akef H, Kotb N, Abo-Elmatty D, Salem S (2017) Anti-proliferative effects of *Androctonus Amoreuxi* scorpion and *Cerastes cerastes* snake venoms on human prostate cancer cells. J Cancer Prev 22(1):40–46. 10.15430/jcp.2017.22.1.40 28382285 10.15430/JCP.2017.22.1.40 PMC5380188

[73] Abdelglil MI, Abdallah SO, El-Desouky MA, Alfaifi MY, Elbehairi SEI, Mohamed AF (2021) Evaluation of the anticancer potential of crude, irradiated *Cerastes cerastes* snake venom and Propolis ethanolic extract & related biological alterations. Molecules 26(22):7057. 10.3390/molecules26227057 34834153 10.3390/molecules26227057 PMC8625720

[74] Badr G, Al-Sadoon MK, Rabah DM, SayedD (2013) Snake (*Walterinnesia aegyptia*) venom-loaded silica nanoparticles induce apoptosis and growth arrest in human prostate cancer cells. Apoptosis 18(3):300–314. 10.1007/s10495-012-0787-1 23238991 10.1007/s10495-012-0787-1

[75] Badr G, Al-Sadoon MK, Rabah DM (2013) Therapeutic efficacy and molecular mechanisms of snake (*Walterinnesia aegyptia*) venom-loaded silica nanoparticles in the treatment of breast cancer- and prostate cancer-bearing experimental mouse models. Free Radic Biol Med 65:175–189. 10.1016/j.freeradbiomed.2013.06.018 23811005 10.1016/j.freeradbiomed.2013.06.018

[76] İlhan S, Çiçek K, Tok CV, Atmaca H (2021) Profiling of apoptosis-associated proteins in human prostate cancer cells in response to *Montivipera bulgardaghica albizona* venom by protein array. Toxin Rev 40(4):1040–1047. 10.1080/15569543.2020.1826970

[77] Harvey AL (2013) Snake Peptides. In Handbook of Biologically Active Peptides, 2nd edition. Elsevier, pp 451–460. 10.1016/B978-0-12-385095-9.00062-2

[78] Mahadevappa R, Ma R, Kwok HF (2017) Venom peptides: improving specificity in cancer therapy. Trends Cancer 3(9):611–614. 10.1016/j.trecan.2017.07.004 28867164 10.1016/j.trecan.2017.07.004

[79] Son DJ, Park MH, Chae SJ, Moon SO et al (2007) Inhibitory effect of snake venom toxin from *Vipera lebetina turanica* on hormone-refractory human prostate cancer cell growth: induction of apoptosis through inactivation of nuclear factor κB. Mol Cancer Ther 6(2):675–683. 10.1158/1535-7163.mct-06-0328 17308063 10.1158/1535-7163.MCT-06-0328

[80] Swaim MW, Chiang HS, Huang TF (1996) Characterisation of platelet aggregation induced by PC-3 human prostate adenocarcinoma cells and inhibited by venom peptides, trigramin and rhodostomin. Eur J Cancer 32(4):715–721. 10.1016/0959-8049(95)00648-6 10.1016/0959-8049(95)00648-6 8695278

[81] Alberghini-dos-Santos JV, Sanchez CA, Bordon KDCF, Pucca MB, Antunes LMG, Arantes EC, de Oliveira IS (2024) Effects of crotamine in human prostate cancer cell line. Toxicon 243:107746. 10.1016/j.toxicon.2024.107746 38704124 10.1016/j.toxicon.2024.107746

[82] Chaisakul J, Hodgson WC, Kuruppu S, Prasongsook N (2016) Effects of animal venoms and toxins on hallmarks of cancer. J Cancer 7(11):1571–1578. 10.7150/jca.15309 27471574 10.7150/jca.15309 PMC4964142

[83] Guo C, Liu S, Yao Y, Zhang Q, Sun MZ (2012) Past decade study of snake venom L-amino acid oxidase. Toxicon 60:302–311 22579637 10.1016/j.toxicon.2012.05.001

[84] Lee ML, Fung SY, Chung I, Pailoor J, Cheah SH, Tan NH (2014) King cobra (*ophiophagus hannah)* venom L-amino acid oxidase induces apoptosis in PC-3 cells and suppresses PC-3 solid tumor growth in a tumor xenograft mouse model. Int J Med Sci 11(6):593–601. 10.7150/ijms.8096 24782648 10.7150/ijms.8096 PMC4003544

[85] Polloni L, Costa TR, Morais LP, Borges BC et al (2023) Oxidative stress induced by Pollonein-LAAO, a new L-amino acid oxidase from *Bothrops moojeni* venom, prompts prostate tumor spheroid cell death and impairs the cellular invasion process *in vitro*. Cell Signal 109:110785. 10.1016/j.cellsig.2023.110785 37364850 10.1016/j.cellsig.2023.110785

[86] Tan KK, Ler SG, Gunaratne J, Bay BH, Ponnampalam G (2017) *In vitro* cytotoxicity of L-amino acid oxidase from the venom of *Crotalus mitchellii pyrrhus*. Toxicon 139:20–30. 10.1016/j.toxicon.2017.09.012 28943466 10.1016/j.toxicon.2017.09.012

[87] Lu W, Hu L, Yang J, Sun X et al (2018) Isolation and pharmacological characterization of a new cytotoxic L-amino acid oxidase from *Bungarus multicinctus* snake venom. J Ethnopharmacol 213:311–320. 10.1016/j.jep.2017.11.026 29180043 10.1016/j.jep.2017.11.026

[88] Samel M, Trummal K, Siigur E, Siigur J (2012) Effect of HUVEC apoptosis inducing proteinase from *Vipera lebetina* venom (VLAIP) on viability of cancer cells and on platelet aggregation. Toxicon 60(4):648–655. 10.1016/j.toxicon.2012.03.023 22781133 10.1016/j.toxicon.2012.03.023

[89] Villarrubia VG, Costa LA, Díez RA (2004) Secreted phospholipases A_2_ (sPLA_2_): friends or foes? Actors of the antibacterial and anti-HIV resistance? Med Clin 123(19):749–757. 10.1016/S0025-7753(04)74656-4 10.1016/s0025-7753(04)74656-4 15574291

[90] Quintana J, Avila I, Ceballos J, Vargas L, Estrada S (2017) Efecto citotóxico de fosfolipasas A_2_ del veneno de *Crotalus durissus cumanensis* de Colombia. Rev Invest Salud 4(1):17–36. 10.24267/23897325.194

[91] Naves MPC, de Morais CR, de Freitas V, Ribeiro DL et al (2021) Mutagenic and genotoxic activities of phospholipase A_2_ Bothropstoxin-I from *Bothrops jararacussu* in Drosophila melanogaster and human cell lines. Int J Biol Macromol 182:1602–1610. 10.1016/j.ijbiomac.2021.05.114 34033823 10.1016/j.ijbiomac.2021.05.114

[92] Tran TV, Siniavin AE, Hoang AN, Le MTT et al (2019) Phospholipase A_2_ from krait *Bungarus fasciatus* venom induces human cancer cell death *in vitro*. PeerJ 7:e8055. 10.7717/peerj.8055 31824756 10.7717/peerj.8055 PMC6896944

[93] Marcinkiewicz C (2013) Applications of snake venom components to modulate integrin activities in cell-matrix interactions. Int J Biochem Cell Biol 45(9):1974–1986. 10.1016/j.biocel.2013.06.009 23811033 10.1016/j.biocel.2013.06.009 PMC3774133

[94] Márquez P (2021) Aislamiento y pruebas de actividad biológica de desintegrinas de *Croatalus polystictus*. Undergraduate Dissertation, Universidad de las Américas Puebla-México

[95] Lin E, Wang Q, Swenson S, Jadvar H et al (2010) The disintegrin contortrostatin in combination with docetaxel is a potent inhibitor of prostate cancer *in vitro* and *in vivo*. Prostate 70(12):1359–1370. 10.1002/pros.21173 20623636 10.1002/pros.21173

[96] Swenson SD, Silva-Hirschberg C, Markland FS (2020) Methods for evaluation of a snake venom-derived disintegrin in animal models of Human Cancer. In: Priel A (ed) Snake and spider toxins. Methods in Molecular Biology, vol 2068. Humana, New York, NY. 10.1007/978-1-4939-9845-6_10 10.1007/978-1-4939-9845-6_10 31576529

[97] Yang RS, Tang CH, Chuang WJ, Huang TH, Peng HC, Huang TF, Fu WM (2005) Inhibition of tumor formation by snake venom disintegrin. Toxicon 45(5):661–669. 10.1016/j.toxicon.2005.01.013 15777962 10.1016/j.toxicon.2005.01.013

